# Antioxidant effects of dexmedetomidine against hydrogen peroxide-induced DNA damage in vitro by alkaline Comet assay

**DOI:** 10.3906/sag-1910-76

**Published:** 2020-08-26

**Authors:** Mustafa S. KOTANOĞLU, Ela KADIOĞLU, Esra EMERCE, etin KAYMAK, Ayşe ZCAN, Hlya BAŞAR

**Affiliations:** 1 Department of Anesthesiology and Reanimation, Ankara Training and Research Hospital, University of Health Sciences, Ankara Turkey; 2 Department of Pharmaceutical Toxicology, Faculty of Pharmacy, Gazi University, Ankara Turkey

**Keywords:** Antioxidant, dexmedetomidine, DNA damage

## Abstract

**Background/aim:**

Dexmedetomidine (DEX) is an alpha-2 adrenergic agonist that is commonly used as a sedative and anesthetic. The protective effects of DEX against oxidative damage under both in vitro and in vivo conditions have been demonstrated. It was aimed to evaluate and compare the protective effects of DEX and vitamin C (Vit C) on DNA against H_2_O_2_-induced DNA damage in human lymphocyte cell cultures in vitro by alkaline Comet assay.

**Materials and methods:**

Lymphocyte cell cultures were divided into 5 groups, as the negative control, solvent control, positive control, hydrogen peroxide (H_2_O_2_; 150 μM) + DEX (1 μM; 2.5 μM; 5 μM), and H_2_O_2_ (150 μM) + Vit C (1 μM; 2.5 μM; 5 μM), and incubated at 37 °C for 1 h. Cell viability was measured using the Trypan blue test. DNA damage was measured using the Alkali Comet Technique and the % percent tail intensity was evaluated. Statistical analysis was performed using 1-way ANOVA and the Tukey multiple comparison test.

**Results:**

It was observed that H_2_O_2_ significantly induced DNA damage in the lymphocytes and this damage was decreased significantly with Vit C and DEX. It was observed that Vit C at doses of 1 μM and 2.5 μM had a significantly stronger antioxidant effect, but there was no significant difference between the antioxidant effects of Vit C and DEX with a dose of 5 μM. The dose of 5 μM DEX was found to be the most effective in reducing oxidative DNA damage.

**Conclusion:**

There is limited data on the protective effects of DEX against oxidative DNA damage. The primary effect might be cytoprotection. The results herein showed that DEX was protective against H_2_O_2_-induced in vitro oxidative DNA damage in lymphocyte cell cultures in a dose-dependent manner. DEX might have a potential therapeutic value in the prevention of oxidative DNA damage in patients.

## 1. Introduction

Dexmedetomidine (DEX) is a highly selective alpha-2 adrenergic receptor agonist that is commonly used in clinical practice as a sedative and anesthetic agent due to its sedative, analgesic, hemodynamic stabilizing, and diuretic effects [1,2]. In addition to its sedative and anesthetic effects, its antiinflammatory and antioxidant effects on vital organs, such as the heart [3,4], lungs [5–7], kidneys [8], spinal cord [9], and brain [10], have been demonstrated. DEX has antiinflammatory and protective effects against oxidative damage that have been shown under both in vitro and in vivo conditions [11,12]. It shows these effects probably by inhibiting the toll-like receptor (TLR) [4,13], suppressing high-mobility group box 1 (HMGB1) factor [14], and inhibiting the nuclear factor (NF)-κB and phosphoinositide-3 kinase (PI3K-) signaling pathway [3,15].

Oxidative stress, which is induced by ischemia, mechanical stress, or toxins, is a condition that results from an imbalance between the production of reactive oxygen species (ROS) and free radicals, as well as inappropriate antioxidant functions. The ROS-induced oxidative stress in cells trigger a mechanism that, through the release of cytochrome c and activation of caspase-3, leads to intrinsic apoptosis. ROS play a critical role in maintaining homeostasis and cell signaling [16]. Hydrogen peroxide (H_2_O_2_), a reactive ROS derivative, is considered to be the radical that is most responsible for oxidative damage. It has been widely used to mimic in vitro oxidative stress in many different cell types [17].

ROS can lead to DNA-strand breaks by loss of DNA bases, known as apurinic/apyrimidinic sites, and inhibits transcription. Moreover, the DNA strand break, an indicator of increased oxidative stress, is a complicated process and it is more likely that the body will tend to make mistakes when it attempts to repair itself [18]. There are many antioxidant and DNA repair systems that protect the organism from undesirable consequences of DNA damage. Although these systems work perfectly throughout life, there may be conditions, such as disease or aging, that lead to increased levels of DNA damage and some external protection, such as vitamin administration, would be needed [19]. Therefore, some medications, such as DEX, which may also have some protective effects aside from their crucial effects, are worth focusing on in terms of patient health.

In the present study, the alkaline Comet assay was used to detect DNA damage in lymphocytes. The alkaline Comet assay, which detects single-strand breaks, as well as alkali-labile sites, has been one of the most popular techniques to detect DNA damage over the recent decades. The Comet assay may be conducted in vitro using single cells from immortalized cell lines or in vivo for any tissue that can be dispersed into a single cell suspension. In the Comet assay, the damaged DNA migrates away from the undamaged DNA-containing nucleoid body, resembling the structure of a Comet during electrophoresis. The percentage of DNA in the tail is directly proportional to the percentage of DNA damage and therefore, could be measured [20]. In this study, it was aimed to evaluate the antioxidant capability of DEX against H_2_O_2_-induced DNA damage in human lymphocyte cell cultures in vitro by alkaline Comet assay. Moreover, this effect was compared with vitamin C (Vit C), which is one of the best-known antioxidants against DNA damage.

## 2. Materials and methods

### 2.1. Chemicals

Vitamin C and DEX were obtained from Redox-C 100 mg/mL, (Bayer, Turkey) and Hipnodex 200 mcg/2 mL (Haver Farma İlaç A.Ş., İstanbul, Turkey), respectively. Other chemicals and reagents used in experiments were commercially purchased from Sigma-Aldrich Corp. (St. Louis, MO, USA).

### 2.2. Sample collection and lymphocyte isolation

This study was approved by the Ethics Committee of the Ankara Health Training and Research Hospital of the University of Health Sciences (Date: 01.08.2018, Approval No.: 052). Peripheral blood from 3 healthy donors (nonsmokers; 29, 33, and 49 years old) was collected after all of the subjects signed an informed consent form and filled out the questionnaire. The questionnaire contained certain information about their demographic characteristics and general health status. Subsequently, lymphocytes were isolated using the density gradient centrifugation technique [21].

### 2.3. Viability test

Prior to initiating the experiments, exposure to H_2_O_2_, Vit C, and DEX was assessed individually on the lymphocytes in terms of cell viability. The final concentrations of the treated lymphocytes were 50, 100, and 150 μΜ for H_2_O_2_, and 1, 2.5, and 5 μΜ for Vit C and DEX. The compounds were dissolved in 0.9% NaCl solution to prepare stock solutions and their diluted solutions. The treated lymphocytes were incubated for 1 h at 37 °C and then the Trypan blue exclusion test was performed [22]. The number of unstained/total cells was determined using a hemocytometer under a light microscope.

### 2.4. Treatments

For determining the DNA damage, lymphocyte cultures were performed in 5 groups comprising the negative control (water), vehicle control (0.9% NaCl), positive control (150 μΜ H_2_O_2_), as well as 1, 2.5, and 5 μΜ of DEX or Vit C together with 150 μΜ of H_2_O_2_, for 1 h at 37 °C. The final concentration of compounds in the medium was adjusted to 1% v/v. Next, 3 independent experiments with the samples from the 3 donors in duplicate were performed. After the incubation process, the lymphocytes were centrifuged for 3 min at 200 g and isolated.

### 2.5. Comet assay

For the detection of DNA damage, the alkaline version of the Comet assay was performed, as described by Singh et al. (1988), with minor modifications [23]. Briefly, 100 μL of treated lymphocytes were mixed with 100 μL of 1% low melting point agarose at 37 °C and was spread to a slide pre-coated with 1% normal melting point agarose and immediately covered with a coverslip. Duplicate slides were made for each sample. After gel solidification, the slides were immersed in lysis solution (2.5 M NaCl, 0.1 M Na2EDTA, 10 mM Tris HCl, pH 10, 1% Triton X-100) and kept at 4 °C overnight. The slides were incubated in a cold electrophoresis solution (0.3 M NaOH, 1 mM EDTA, pH > 13) for 20 min to allow DNA unwinding. Next, electrophoresis was performed at 4 °C, 25 V, and 300 mA for 20 min. After that, the slides were washed with neutralization buffer (0.4 M Tris, pH of 7.5) and stained with ethidium bromide (20 μg/mL). A total of 300 cells were randomly selected per treatment and examined using a fluorescence microscope (Zeiss Axioscope, Germany) at 400× magnification for image analysis (Comet assay III image analysis system (Perceptive Instruments, UK). To determine the DNA damage, the percent tail intensity (also known as % tail DNA) was used.

### 2.6. Statistical analysis

Data on viability were displayed as a percentage of the control that was not exposed to H_2_O_2_, Vit C, or DEX. Values are the mean ± SEM of 3 independent experiments. Statistical significance was determined by 1-way ANOVA followed by the the Tukey-Kramer multiple comparison test using GraphPad Prism version 7 (demo version). P < 0.05 was considered statistically significant.

## 3. Results

### 3.1. Viability test

**Figure 1:**
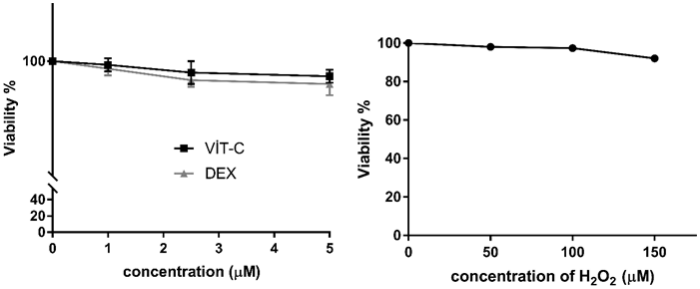
Effects of the H_2_O_2_, Vit C, and DEX treatments for 1 h on the viability % of the human lymphocytes

Figure 1 shows the effects of H_2_O_2_, Vit C, and DEX on the lymphocyte viability. In the Trypan blue test performed to determine whether the Vit C and DEX substances cause a cytotoxic effect on the lymphocytes, viability was found to be above 90% in all of the tested concentrations.

### 3.2. Detection of DNA damage

It was observed that H_2_O_2_ significantly induced DNA damage in the lymphocytes and this damage decreased significantly with Vit C and DEX. When H_2_O_2_ alone was assessed in the 1-h treatments, the H_2_O_2_-induced DNA damage exhibited a dose-dependent response (data not shown). Thus, 150 μM H_2_O_2_, which was not cytotoxic but induced significantly DNA damage, was adopted for the main experiments.

**Figure 2:**
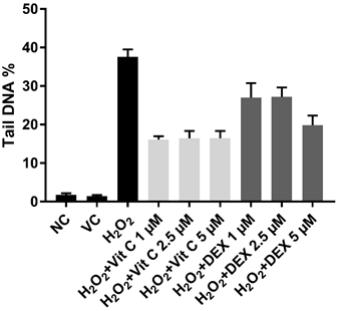
Vit C and DEX antigenotoxic effects on the lymphocytes incubated with H_2_O_2_.

The effect of DEX and Vit C in the lymphocytes cells was demonstrated using various concentrations (1, 2. 5, and 5 μM). Lymphocytes incubated with H_2_O_2_ + Vit C and H_2_O_2_ + DEX at different concentrations showed significantly decreased DNA damage of up to 50% when compared to H_2_O_2_ alone (P < 0.05) (Table). Although Vit C and DEX caused an antigenotoxic effect on the DNA damage, they were not dose-dependent. It was observed that concentrations of 1 and 2.5 μM Vit C were more effective than DEX at the same concentrations. On the other hand, DEX showed a similar antigenotoxic effects on the lymphocytes at a concentration of 5 μM when compared to 5 μM Vit C (P = 0.946) (Figure 2).

**Table:** Protective effects of Vit C and DEX on H_2_O_2_-induced DNA damage on human lymphocytes. NC = Negative control; VC = Vehicle control; *Compared to VC; #Compared to H_2_O_2_.

Treatments	Tail DNA % (mean SEM)	P-value*	P-value^#^
NC	1.75 0.43	>0.9999	
VC	1.38 0.35		
H_2_O_2_	37.55 1.96	<0.0001	
H_2_O_2_+Vit C 1 ?M	16.12 0.86	0.0003	<0.0001
H_2_O_2_+Vit C 2.5 ?M	16.45 1.92	0.0002	<0.0001
H_2_O_2_+Vit C 5 ?M	16.46 1.91	<0.0001	<0.0001
H_2_O_2_+DEX 1 ?M	27.04 3.75	<0.0001	0.0202
H_2_O_2_+DEX 2.5 ?M	27. 2.45	<0.0001	0.0230
H_2_O_2_+DEX 5 ?M	19.84 2.53	<0.0001	<0.0001

## 4. Discussion

In general, oxidative DNA damage induces cytotoxicity, leading to multiple organ damage, which may result in multisystem organ failure. Oxidative DNA damage is known to directly induce cytotoxicity and can also alter cell signaling pathways. Interestingly, the source of oxidative injury may be a key to the extent of cellular cytotoxicity [24]. The overwhelming production of oxidative injury threatens the integrity of protein oxidation and leads to DNA strand breaks, resulting in tissue damage [25]. The aim of this study was to investigate the effects of the alpha-2 adrenoceptor agonist, DEX, against H_2_O_2_-induced oxidative DNA damage in vitro.

Mechanistically, it was previously reported that DEX acts as an antiinflammatory agent and provides cell protection by increasing the expression of cell survival proteins and reducing apoptosis. In addition, DEX has a structure similar to imidazoline and its antiapoptotic effect is enhanced by the activation of imidazoline receptors [26]. It has been demonstrated that TLR4 and NF-𝜅B signaling is involved in the DEX-mediated protection against oxidative injury. Gao et al. used TLR4 knockdown by TLR4-RNA transfection and overexpression by TLR4-DNA transfection in vitro approaches to explore the mechanisms underlying DEX-mediated protection [4]. The expression of TLR4 has been shown to be triggered through endogenous ligands, including damageassociated molecular patterns and cytokines. Terminal deoxynucleotidyl transferase-mediated digoxigenin deoxyuridine nick-end labeling, which is increased oxidative damage staining to detect dead cells [5]. As a result, DEX may have prevented the increased expression of TLR4 by attenuating tissue injury and the pretreatment with DEX resulted in almost complete attenuation of the TLR4 expression associated with decreased cell death of epithelial cells [4,5].

It has been suggested that human fetal osteoblast cells pretreated with DEX could be protected against H_2_O_2_- induced oxidative stress [27]. Cui et al. demonstrated that DEX attenuated the bilirubin-induced injury of epithelial alveolar cells, both in vitro and in vivo. In this condition, it was described as reducing the alveolar damage and epithelial cell proliferation via its inhibitory effect on bilirubin induced cell cycle arrest [7]. Moreover, it was reported that astrocytes treated with DEX had significantly increased neurotrophic factor production and were shown to preserve cell viability via the release of neurotrophic factors when compared to the control [9]. Although the antiapoptotic and antiinflammatory effects of DEX have been reported to be associated with phosphoinositide kinase and extracellularly signal-regulated kinase signaling pathways [4,5,9,12,28], there is limited data on its protective effects against oxidative DNA damage. In the present study, the antioxidant potential of DEX was investigated and this effect was compared with Vit C in an in vitro model by Comet assay. The results showed that DEX might be a protective agent against H_2_O_2_-induced oxidative DNA damage in lymphocyte cell cultures in vitro. Therefore the primary effect of DEX might be cytoprotection. This allows DEX to act as an antioxidant against oxidative DNA damage following H_2_O_2_ administration. A concentration of 5 μM DEX was found to be the most effective in reducing oxidative DNA damage. Although it began at higher doses, this effect was comparable with that of Vit C.

The protective effect of DEX, which is comparative to ahighly effective antioxidant like Vit C, might be associated either with phosphoinositide kinase and extracellularly signal-regulated kinase signaling pathways or alteration in transcription factor control. The mechanisms for altered transcription factor control could either be via decreased binding to promoter regions via oxidative damage to DNA or more directly by redox regulation of transcription factor activation [29] and/or altered DNA-binding due to redox-induced modification of the transcription factor protein [30].

On the other hand, HMGB1, which is strong damageassociated molecular pattern released from dying cells during oxidative damage, acts by binding to TLR-4 to initiate the downstream NF-B signaling cascade that greatly increases the synthesis of proinflammatory cytokines [31]. HMGB1 proteins are targeted to particular DNA sites in chromatin by either protein-protein interactions or recognition of specific DNA structures. Furthermore, the accumulation of HMGB1 protein is found at sites of oxidative DNA damage in live cells, thus defining HMGB1 as a component of an early DNA damage response [14]. As shown in a previous study, DEX caused reduced translocation of HMGB1 from the nucleus to the cytoplasm. HMGB1 levels in the nucleus were significantly reduced after oxidative damage, but the addition of DEX significantly increased the HMGB1 protein levels in the cytoplasm [14].

In conclusion, the results showed that in vitro oxidative DNA damage in lymphocyte cell cultures can be prevented by DEX administration. DEX showed protective effects against H_2_O_2_-induced DNA damage in vitro and this effect was comparable with that of Vit C, which is a known antioxidant. It is therefore suggested that DEX might have a potential therapeutic value in the prevention of oxidative DNA damage in patients.

## Acknowledgement/disclaimers

The present study was financially supported by the Ankara Research and Training Hospital Research Fund under grant number 0056 on October 3rd, 2018.
